# Cardiometabolic risk is unraveled by color Doppler ultrasound of the clitoral and uterine arteries in women consulting for sexual symptoms

**DOI:** 10.1038/s41598-021-98336-7

**Published:** 2021-09-22

**Authors:** I. Scavello, E. Maseroli, S. Cipriani, V. Di Stasi, N. Verde, D. Menafra, S. Scannerini, S. Marchiani, G. Rastrelli, V. Ricca, F. Sorbi, M. Fambrini, F. Petraglia, M. Maggi, Linda Vignozzi

**Affiliations:** 1 Department of Experimental Clinical and Biomedical Sciences “Mario Serio”, University of Florence, Viale Pieraccini 6, 50134 Florence, Italy; 2 Andrology, Women’s Endocrinology and Gender Incongruence Unit, Azienda Ospedaliero-Universitaria Careggi, Florence, Italy; 3 Clinical Medicine and Surgery Department, Section of Endocrinology, Unit of Andrology, Reproductive Medicine and Male and Female Sexuality (FERTISEXCARES), Federico II University of Naples, Naples, Italy; 4 Psychiatric Unit, Department of Neuroscience, Psychology, Drug Research and Child Health, University of Florence, Florence, Italy; 5 Department of Experimental and Clinical Medicine, University of Florence, Florence, Italy; 6 Gynecology Unit, Department of Biomedical, Experimental and Clinical Sciences “Mario Serio”, University of Florence, Florence, Italy; 7 Endocrinology Unit, Department of Experimental Clinical and Biomedical Sciences “Mario Serio”, University of Florence, Florence, Italy

**Keywords:** Hormones, Endocrinology, Endocrine reproductive disorders, Gonadal disorders, Circulation, Metabolism, Human behaviour

## Abstract

Female sexual dysfunction (FSD) may be a mirror of a poor cardiometabolic state. In a small pilot study enrolling 71 women with FSD, we previously demonstrated that clitoral Pulsatility Index (PI) evaluated by using color Doppler ultrasound (CDU), reflecting vascular resistance, was associated with cardiometabolic risk factors. Data on uterine CDU in this context are lacking. First, to confirm previously reported data on the direct association between clitoral PI and cardiometabolic risk factors on a larger study population of women consulting for sexual symptoms; second, to investigate eventual similar correlations between cardiometabolic risk factors and CDU parameters of the uterine artery. We also ascertained whether uterine artery PI, similarly to what had previously been observed for clitoral artery PI, was directly related to body image uneasiness and psychopathological symptoms, assessed by validated questionnaires. N = 230 women consulting our clinic for sexual symptoms were examined with clitoral CDU and blood sampling and were asked to fill out the Female Sexual Function Index, the Middlesex Hospital Questionnaire (MHQ) and the Body Uneasiness Test (BUT). In a subgroup of women (n = 164), we also performed transvaginal CDU with measurement of uterine artery parameters. At multivariate analysis, we found a direct association between clitoral PI and body mass index (BMI) (*p* = 0.004), waist circumference (WC) (*p* = 0.004), triglycerides (*p* = 0.006), insulin (*p* = 0.029) and HOMA-IR (*p* = 0.009). Furthermore, a correlation between obesity and Metabolic Syndrome (MetS) and a higher clitoral PI was observed (*p* = 0.003 and *p* = 0.012, respectively). Clitoral PI was also correlated with MHQ-S (*p* = 0.010), a scale exploring somatized anxiety symptoms, and BUT-B Positive Symptom Distress Index (*p* = 0.010), a measure of body image concerns. Similarly, when investigating the uterine artery, we were able to demonstrate an association between its PI and BMI (*p* < 0.0001), WC (*p* = 0.001), insulin (*p* = 0.006), glycated haemoglobin (*p* =  < 0.0001), and HOMA-IR (*p* = 0.009). Women diagnosed with obesity and MetS showed significantly higher uterine PI values vs. those without obesity or MetS (*p* = 0.001 and *p* = 0.004, respectively). Finally, uterine PI was associated with BUT-A Global Severity Index (*p* < 0.0001) and with several other BUT-A subdomains. Vascular resistance of clitoral and uterine arteries is associated with cardiometabolic risk factors and body image concerns in women consulting for sexual symptoms. If further confirmed in different populations, our data could suggest CDU, a common examination method, as a useful tool for an identification—and possible correction—of cardiometabolic risk factors.

## Introduction

A gender disparity related to the relationship between sexual dysfunction and cardiovascular (CV) risk still endures. It is well known that erectile dysfunction (ED) is a sign of subclinical CV pathology and that alterations in penile color Doppler ultrasound (CDU) parameters are a predictive marker of major CV events^1^.

Several studies have shown an increased prevalence of female sexual dysfunction (FSD) in women with metabolic alterations, in particular when clustered in the metabolic syndrome (MetS)^2–7^. Therefore, it was hypothesised that an impairment of genital vascularization could be a harbinger for CV diseases also in women^8^. Inadequacy of the methodologic approach used to investigate cardiometabolic risk in patients with FSD has accounted for this gender gap so far. Indeed, unlike penile CDU^9–11^, clitoral CDU has only recently come into the spotlight as a promising technique for the objective assessment of vasculogenic FSD^12^. This refers to “vaginal engorgement insufficiency” and “clitoral erectile insufficiency”, two conditions resulting from the alteration of the hemodynamic responses to sexual stimulation in women with CV risk factors^13^.

In this context, we previously conducted a proof-of-concept pilot study, analysing the clinical correlates of clitoral CDU parameters in 71 women with FSD^12^. Essentially, we demonstrated that the different components of MetS were independently associated with the increase of the clitoral pulsatility index (PI), an index of vascular resistance, which in turn was correlated with a reduction in sexual arousal.

Hitherto, the CDU assessment of the uterine artery has been used in the obstetrical-gynecological field in the evaluation of foetal umbilical vessel flow and foetal circulation^14,15^, and in uterus-placental studies to predict foetal growth-related delays^16^. In assisted reproductive technology (ART), uterine artery resistance is as a good indicator of the probability of pregnancy^17^. No data are available on the association between uterine artery resistance and CV risk in women with sexual symptoms.

The aim of this study was to consolidate our data on the direct correlation between clitoral PI, assessed by CDU, and CV risk factors^12^, using a large sample of women consulting for sexual difficulties. In addition, in a subgroup of FSD patients, the uterine artery PI has been evaluated, in order to investigate whether, analogously to the clitoral artery, there could be a direct correlation with cardiometabolic risk factors. We also ascertained whether uterine artery PI, similarly to what was previously observed for clitoral artery PI, was directly related to body image uneasiness and psychopathological symptoms, which are crucial variables to evaluate in studies on FSD^12,18^. Our hypothesis was that vascular resistance not only in the clitoral arterial district, but also in the uterine one, could be directly associated with worse cardiometabolic and psychosexual profiles in women with sexual symptoms.

## Methods

230 female pre- and post-menopausal patients referring to our Andrology, Women’s Endocrinology and Gender Incongruence Unit for sexual symptoms were consecutively recruited. Women were admitted by self-referral or referred by their general practitioners or other specialists (i.e. urologist, oncologist, neurologist, diabetologist, etc.). Inclusion criteria were being sexually active in the previous 4 weeks and having a partner. In particular, the presence of a relationship was investigated by a specific question included in an interview administered during the visit: “Do you have a stable relationship with a partner? Do you live together?”, with scoring 0 = Stable relationship, living together; 1 = Stable relationship, not living together; 2 = No stable relationship; 3 = No relationship^19^. Only women scoring 0 to 2 were included in the analysis.

Clinical data were collected during outpatient visits, after administration of an informed consent. The study protocol was designed according to the Helsinki Declaration and approved by the local ethics committee (protocol 37.589/SPE. 13.034, Comitato Etico Area Vasta Centro CEAVC).

During the first visit, demographic, physiological and pathological data were collected, and body mass index (BMI), waist circumference (WC) and systolic (SBP) and diastolic blood pressure (DBP) were measured.

### Psychometric assessment

Standardized questionnaires relative to sexual and psychological health were administered, including the Female Sexual Function Index (FSFI)^20^, the Female Sexual Distress Scale-Revised (FSDS-R)^21^, the Body Uneasiness Test (BUT)^22^ and the Middlesex Hospital Questionnaire (MHQ)^23^. The average time taken to fill the questionnaires was around 20 min. To avoid distractions, participants completed the questionnaires alone, in a quiet context.

The FSFI is the most common questionnaire used for the screening of FSD and consists of 6 domains investigating different phases of the female sexual response (desire, arousal, orgasm), sexual satisfaction and dyspareunia^20^. The score of each item ranges from a minimum of 0/1 to a maximum of 5 and is obtained by the addition of the single question score multiplied by a specific factor. The total score is the result of the 6 score items’ addition. The patient with a total score ≤ 26.55 is suffering from FSD. The Desire domain is the only domain that can be used independently^24^, and a cut-off score of 5 has been found to identify women with Hypoactive Sexual Desire Disorder with a good specificity and sensibility^25^.

The FSDS-R is validated to assess sex-related distress^21^.

The BUT is used to assess body image concerns and eventual related pathological conditions. It includes two different parts: the first part (BUT-A) consists of questions regarding body-related attitudes, while the second part (BUT-B) explores the level of dissatisfaction towards 37 different body parts considered separately^22^. BUT-A total score indicates global dissatisfaction with body image and is expressed as Global severity index (GSI), which is considered as pathological if > 1.2; BUT-A subscales define weigh-related phobia (Weight Phobia, BUT-WP), avoiding attitudes (Avoiding, BUT-AV), compulsive control of self-image (Compulsive self-monitoring, BUT-CSM), tendency towards separation from one’s own body (Depersonalization, BUT-D), and concern about body image (BUT-BIC, Body image concerns)^22^. A 6-point score on a Likert scale (with answers ranging from “never” to “always”) is attributed to every question, with the higher scores corresponding to a more severe discomfort^22^. BUT-B score indicates dissatisfaction with a single body part and is expressed in two global measures: a global dissatisfaction index (Positive Symptom Total, PST) and a discomfort index (Positive Symptom Distress Index, PSDI)^22^.

The MHQ-modified is generally used in non-specialistic settings to evaluate psychopathological traits and it includes a total score and 6 different subscales: free anxiety symptoms (MHQ-A), phobic anxiety symptoms (MHQ-P), obsessive–compulsive traits and symptoms (MHQ-O), somatized anxiety symptoms (MHQ-S), depressive symptoms (MHQ-D) and hysteric/histrionic symptoms (MHQ-H)^23^.

### Biochemical evaluation

Blood samples were drawn in the morning, after an overnight fast, for determination of the biochemical parameters considered necessary for the evaluation of FSD. In particular, the assessed parameters were: blood glucose (esokinase method; Dimension Vista 1500 Medical Solutions by Siemens Healthcare, Newark, USA); total and high-density lipoprotein (HDL) cholesterol (high density lipoprotein) and triglycerides (automatic enzymatic colorimetric method; Dimension Vista 1500 Medical Solutions by Siemens Healthcare, Newark, USA); insulin (electrochemiluminescence immunoassay, “ECLIA”; Roche Diagnostics, Mannheim, Germania) and glycated haemoglobin (HbA1c) (high prestations liquid chromatography, HPLC, Variant II method, Biorad Laboratories, Hercules, CA, USA). LDL (low-density lipoprotein) cholesterol was calculated through Friedewald equation: LDL cholesterol = total cholesterol—(HDL cholesterol + triglycerides/ 5) and all parameters were expressed in mg/dl. HOMA-index (Homeostatic Model Assessment), a method used to estimate insulin-resistance (IR) and beta-cell function, was calculated according to the following formula: HOMA-IR = (glycaemia x insulin)/22.5 (see http://www.phc.ox.ac.uk/research/tech-nology-outputs/ihoma2).

### Metabolic syndrome (MetS) diagnosis

MetS diagnosis was defined according to the National Cholesterol Education Program—Third Adult Treatment Panel (NCEP-ATPIII; Expert Panel on Detection, Evaluation, and Treatment of High Blood Cholesterol in Adults, 2001), in the presence of ≥ 3 of the following factors: central obesity (waist circumference ≥ 88 cm), high triglyceride serum levels (≥ 150 mg/dL or specific therapy), arterial hypertension (systolic blood pressure ≥ 130 mmHg and/or diastolic blood pressure ≥ 85 mmHg or specific therapy), impaired fasting glycaemia (≥ 110 mg/dL or specific therapy) and low HDL cholesterol serum levels (< 50 mg/dL or specific therapy).

### Instrumental investigations

All patients (n = 230) underwent color Doppler ultrasound (CDU) with examination of the clitoral cavernous artery, in order to measure its Pulsatility Index (PI). In fertile women, CDU was performed in the follicular phase of the menstrual cycle (days 3–5). The phase of the menstrual cycle was registered during the visit as reported by the patient and later confirmed by the ultrasound measurement of endometrial thickness. The evaluation was performed by an experienced operator, blinded to other clinical data, with a linear probe (LA523, 6–13 MHz) using a MyLabClass-C ultrasound system (Esaote S.p.A., Genoa, Italy). In order to reduce the impact of external factors on blood flow, the patients were examined in a quiet environment, with constant temperature and lighting. To minimize the risk of situational anxiety altering the data due to vasoconstriction, only one operator stayed in the room, and the exam was carried out following a clear and complete explanation. The procedure follows that the one previously published by Battaglia and collaborators^26–29^.

To standardize operating conditions, all patients were instructed to refrain from sexual intercourse and masturbation for at least 12 h. CDU was performed in a gynecological position and with an empty bladder. An adequate amount of ultrasound gel was used in order to avoid air interference, and to prevent artifacts, a slight pressure on the genitals was exerted. The transverse scan of the clitoris was obtained by positioning the probe transversely, on the upper part of the vulva; this projection allows operators to easily locate the cavernous arteries, which appear well defined in the centre of the two cavernous bodies. When an adequate signal was detected, the pulsed-wave Doppler mode, which records the velocity of blood flow, was activated. Clitoral PI is automatically calculated and represents the difference between the systolic peak velocity and the end-diastolic velocity divided by the average velocity^30^; since it characterizes the shape of the wave, the PI is independent of the angle between the probe and the vessel^31^. For each cavernous artery, at least 3 waves of similar shape were sampled, and the mean PI was calculated. No significant difference was observed between the left and the right clitoral artery PI.

In addition, in a subgroup of patients (n = 164), transvaginal CDU was performed to evaluate the uterine artery PI. Since several factors, common in pre-menopausal women, can influence uterine vascularization (i.e. the phase of the menstrual cycle, endometriosis, organic disorders including fibroids, puerperium or recent abortion), we analysed the sample according to patients’ pre- (n = 99) or post-menopausal (n = 65) status. All patients were evaluated at the same time of the day, to avoid flow changes due to the circadian rhythm. The examination was performed in a dorsal lithotomy position. The measurement was performed on the right side, lateral to the cervix at the internal orifice, before the branching of the artery. Three surveys were carried out in a time window of 1–3 min to define a mean value.

### FSD work-up

After recruitment, all participants underwent a complete diagnostic work-up performed by a multidisciplinary team, involving an endocrinologist, a gynaecologist and a mental health professional (psychiatrist and/or psychologist) all trained in sexual medicine. As per clinical practice, after FSD diagnosis, and following a complete evaluation of their personal, relational and social background, needs, preferences, and contraindications, women could be offered medical (i.e. local treatment for vulvo-vaginal atrophy, off-label systemic testosterone treatment) and/or psychological treatment (i.e. cognitive behavioral therapy, sexual therapy). The partner was not directly involved in the initial assessment, but the possibility of his involvement was offered to the patient during the following visits.

### Statistical analyses

Data were expressed as mean ± standard deviation when normally distributed, as median (interquartile range) when non-normally distributed, and as percentage when categorical. When significant differences among groups were found, the unpaired 2-sided Student t test was used for comparisons between 2 groups. For non-normally distributed parameters, comparison between groups of variables was also performed with non-parametric tests (Mann Whitney U test for comparisons between 2 groups). Correlations between variables were evaluated through Spearman method and ANCOVA with post-hoc Bonferroni test was used for differences between groups. Finally, linear or ordinal regression was used for multivariate analysis, considering confounding factors as specified. All statistical analyses were performed using SPSS for Windows 25.0 (IBM, Armonk, NY, USA). In order to consider the multiple comparisons problem, we performed a False Discovery Rate Correction. After 10% FDR calculation, we adjusted the cut-offs for significant *p*-value at < 0.038.

### Ethical approval

The study protocol was designed according to the Helsinki Declaration and approved by the local ethics committee (Comitato Etico Area Vasta Centro).

### Informed consent

Informed consent was obtained from all individual participants included in the study.

## Results

### Characteristics of the sample

Table 1 shows the main clinical, metabolic, and psycho-sexual characteristics and the mean clitoral pulsatility index (PI) of the total sample (n = 230). Psycho-sexual parameters were assessed by using FSFI, FSDS-R, MHQ and BUT questionnaires. The same characteristics are also reported according to menopausal status (Supplementary Table 1a and 1b; post-menopausal n = 114; pre-menopausal n = 116). After adjusting for age, post-menopausal women showed an older age, a higher number of parities, a more frequent history of breast or pelvic surgery and a worse metabolic profile compared to pre-menopausal ones (Supplementary Table 1a). Concerning the psychosexual questionnaires, in an age-adjusted model, post-menopausal women only showed lower FSFI total and desire domain scores (Supplementary Table 1b). In contrast, clitoral PI showed no statistically significant difference between post-menopausal and premenopausal women (Supplementary Table 1b).

**Table 1:** Baseline characteristics of the sample (N = 230): clinical history, metabolic parameters, psycho-sexual parameters and CDU parameters.

	N = 230 women
**Clinical history**	
Age (years)	43.1 ± 12.9
Menopause, % (n)	49.9% (114)
Menopause, Surgical, % (n)	5.0% (6)
Stable relationship, % (n)	89.9% (207)
Current smoking habit, % (n)	19.0% (44)
Physical activity, % (n)	33.8% (78)
Parity, % (n)	14.3% (33)
Waist circumference (cm)	93.1 ± 16.6
BMI (kg/m^2^)	24.9 ± 6.1
Cardiovascular diseases, % (n)	3.0% (7)
Diabetes mellitus, % (n)	4.3% (10)
Dyslipidemia, % (n)	15.8% (36)
Hypertension, % (n)	18.5% (43)
Specific medications	
Hypoglycemic drugs, % (n)	6.7% (15)
Lipid-lowering drugs, % (n)	5.8% (13)
Antihypertensive drugs, % (n)	15.4% (35)
Psychiatric drugs, % (n)	23.3% (53)
Urinary or gynecologic infections (actual or in the past 3 months), % (n)	56.5% (130)
Urinary or gynecologic diseases and infections (in the past), % (n)	52.2% (120)
Endometriosis, % (n)	6.1% (14)
PCOS, % (n)	5.5% (13)
Oral Contraception, % (n)	16.9% (39)
Hormonal Replacement Therapy, % (n)	9.2% (21)
Pelvic Surgery, % (n)	26.9% (62)
Breast Surgery, % (n)	8.6% (20)
Other Surgery , % (n)	30.0% (69)
Oncologic diseases, % (n)	11.7% (30)
Breast Cancer, % (n)	1.6% (4)
Psychiatric diseases, % (n)	33.3% (77)
Neurological diseases, % (n)	1.8% (4)
**Metabolic parameters**	
Systolic blood pressure (mm Hg)	120.00 [110.00–130.00]
Diastolic blood pressure (mm Hg)	75.00 [70.00–80.00]
Fasting glucose (g/L)	0.90 ± 0.14
Fasting insulin (mU/L)	9.20 ± 8.91
HbA1c (mmol/mol)	36.01 ± 5.48
Total Cholesterol (mg/dl)	201.90 ± 38.08
HDL Cholesterol (mg/dl)	63.65 ± 15.59
LDL Cholesterol (mg/dl)	119.38 ± 32.37
Triglycerides (mg/dl)	80.00 [60.25–112.00]
**Psycho-sexual parameters**	
FSFI Total score	20.6 [11.9–26.3]
FSFI Desire	2.4 [1.8–3.6]
FSFI Arousal	2.7 [1.5–4.5]
FSFI Lubrication	3.6 [1.5–5.4]
FSFI Orgasm	3.6 [1.2–5.2]
FSFI Satisfaction	3.6 [2.0–5.2]
FSFI Pain	3.6 [1.2–6.0]
FSFI pathological total score % (n)	92.2% (212)
MHQ Total score	35.5 [26.0–46.0]
FSDS-R score	19.0 [7.0–33.0]
FSDS-R pathological score % (n)	64.8% (149)
FSFI and FSDS-R pathological scores % (n)	58.3% (134)
BUT-A global severity index (GSI)	0.7 [0.4–1.5]
BUT-A weight phobia (WP)	1.1 [0.5–2.4]
BUT-A body image concern (BIC)	1.0 [0.5–1.9]
BUT-A avoidance (AV)	0.2 [0.0–0.8]
BUT-A compulsive self-monitoring (CSM)	0.6 [0.2–1.2]
BUT-A depersonalization (DEP)	0.3 [0.0–0.8]
BUT-B positive symptom Total (PST)	9.0 [5.0–17.0]
BUT-B positive symptom distress index (PSDI)	2.0 [1.6–1.8]
**CDU parameters**	
Clitoral PI	1.6 ± 07

Data are expressed as mean ± SD when normally distributed, median (quartile) when not normally distributed, and percentage when categorical.

*BMI* body mass index, *Hba1c* glycated hemoglobin, *HDL* high-density lipoprotein, *LDL* low-density lipoprotein, *PCOS* polycystic ovary syndrome, *CDU* color Doppler ultrasound, *PI* pulsatility index.

### Correlation between clitoral PI and cardiometabolic/psychosexual parameters

We next evaluated the correlations between the clinical and biochemical features and clitoral PI. At univariate analysis, and after multivariate adjustment for confounders (age, smoking habit, and years since menopause), clitoral PI showed a positive correlation with BMI, WC, triglyceride levels, insulin levels and HOMA index (Table 2).

**Table 2:** Associations between clitoral pulsatility index (PI) and metabolic and psychological parameters (n = 230).

	Clitoral PI			
	Univariate analysis		Multivariate analysis*	
	Pearson’s r	*P*	β	*P*
**Clinical and metabolic parameters**				
BMI (kg/m2)	0.200	**0.002**	0.215	**0.004**
Waist circumference (cm)	0.214	**0.003**	0.240	**0.004**
SBP (mm Hg)	0.142	0.043	1.055	0.293
DBP (mm Hg)	0.011	0.876	− 0.052	0.533
Fasting glycemia (g/L)	0.062	0.380	0.056	0.490
Total cholesterol (mg/dL)	0.121	0.085	0.163	0.069
HDL cholesterol (mg/dL)	− 0.045	0.519	− 0.088	0.285
Triglycerides (mg/dL)	0.191	**0.006**	0.220	**0.006**
LDL cholesterol (mg/dL)	0.109	0.129	0.157	0.068
Fasting insulin (mU/L)	0.193	**0.027**	0.233	**0.029**
HOMA-IR (units)	0.199	**0.033**	0.293	**0.009**
HbA1c (mmol/mol)	0.009	0.909	− 0.031	0.753
**Psychological parameters**				
MHQ Total Score	0.082	0.282	0.129	0.123
MHQ Free-floating anxiety symptoms	0.040	0.597	0.084	0.312
MHQ Phobic anxiety symptoms	0.058	0.443	0.071	0.394
MHQ Obsessive–compulsive	0.027	0.722	0.070	0.401
MHQ Somatized anxiety symptoms	0.176	**0.021**	0.217	**0.010**
MHQ Depressive symptoms	0.096	0.206	0.142	0.090
MHQ Hysterical symptoms	− 0.019	0.802	− 0.004	0.961
BUT-A global severity index (GSI)	− 0.048	0.536	0.008	0.923
BUT-A weight phobia (WP)	− 0.067	0.392	0.004	0.967
BUT-A body image concern (BIC)	0.006	0.941	0.065	0.458
BUT-A avoidance (AV)	− 0.044	0.573	− 0.038	0.666
BUT-A compulsive self-monitoring (CSM)	− 0.102	0.189	− 0.050	0.564
BUT-A depersonalization (DEP)	− 0.053	0.500	− 0.016	0.859
BUT-B positive symptom distress index (PSDI)	0.155	0.050	0.225	**0.010**
BUT-B positive symptom Total (PST)	− 0.068	0.396	− 0.066	0.461

*Data adjusted for age, smoking habit, and years since menopause. After 10% False Discovery Rate calculation, the cut-off for significant *p*-value was adjusted at < 0.038. Bold indicates statistical significance (*p* value < 0.038).

*BMI* body mass index, *SBP* systolic blood pressure, *DBP* diastolic blood pressure, *HDL* high-density lipoprotein, *LDL* low-density lipoprotein, *HOMA-IR* homeostasis model assessment—insulin resistance, *HbA1c* glycated hemoglobin, *MHQ* Middlesex Hospital Questionnaire, *BUT* Body Uneasiness Test.

**Figure 1:**
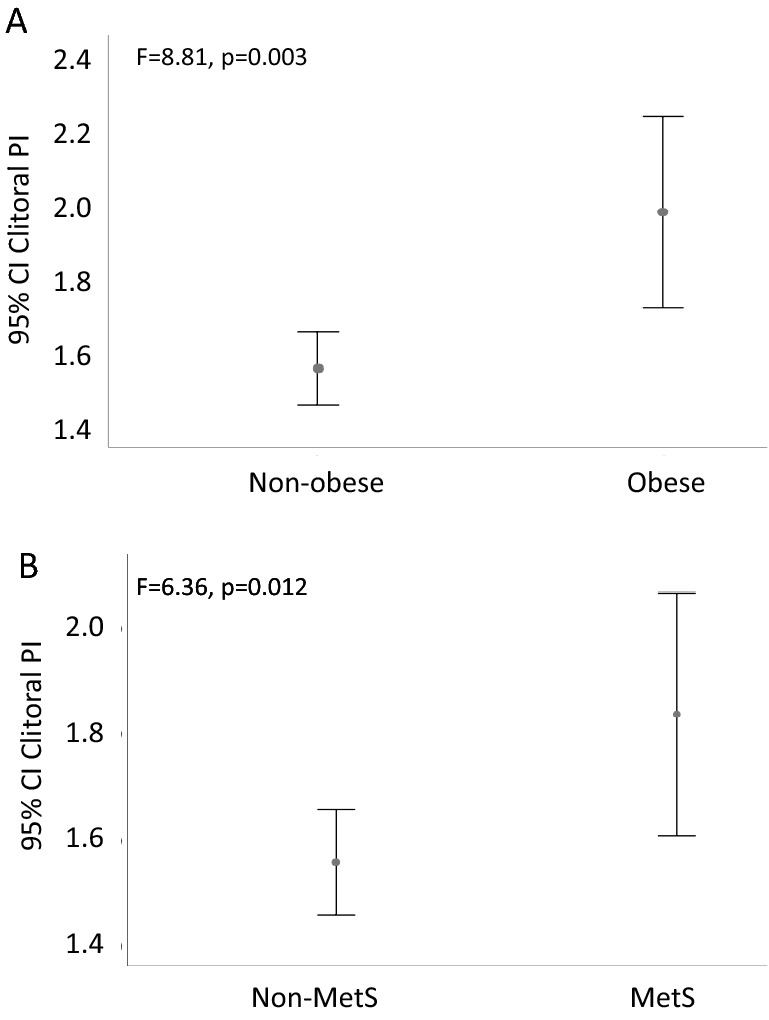
Differences in clitoral artery pulsatility index (PI) in women with FSD according to obesity (BMI ≥ 30 kg/m2, panel **A**) and metabolic syndrome (MetS, panel **B**). Data were adjusted for age, years of menopause and smoking habit. BMI = body mass index.

At analysis of covariance, women with obesity (BMI ≥ 30 kg/m^2^) or MetS (owing ≥ 3 MetS factors) showed significantly higher clitoral PI values as compared to non-obese subjects or to women without MetS (F = 8.962, *p* = 0.003; F = 6.281, *p* = 0.013, respectively). These differences retained statistical significance after adjusting for confounders (age, years since menopause and smoking habit) (F = 8.814, *p* = 0.003 for obesity, Fig. 1A; F = 6.361, *p* = 0.012 for Mets, Fig. 1B).

Considering sexual and psychological parameters, no significant associations were observed between clitoral PI and FSFI or FSDS-R scores (not shown). In contrast, clitoral PI showed significant and positive associations with the MHQ item exploring somatized anxiety symptoms and with BUT-B Global measure PSDI, a score related to global distress for body parts, at either univariate or multivariate analysis (Table 2). Interestingly, when the 37 body parts explored by BUT-B were evaluated (not shown), increased clitoral PI only showed a significant positive association with the item exploring dissatisfaction for the genitals, even in the fully adjusted model (β = 0.169; *p* = 0.05).

### Correlation between uterine PI and cardiometabolic/psychosexual parameters

In a subset of patients (n = 164), CDU of the clitoris was followed by transvaginal ultrasound, thus allowing us to assess also the uterine artery PI. Baseline characteristics of this subgroup, either considered as a whole or after stratification according to the menopausal status, are reported in Supplementary Table 2.

**Figure 2:**
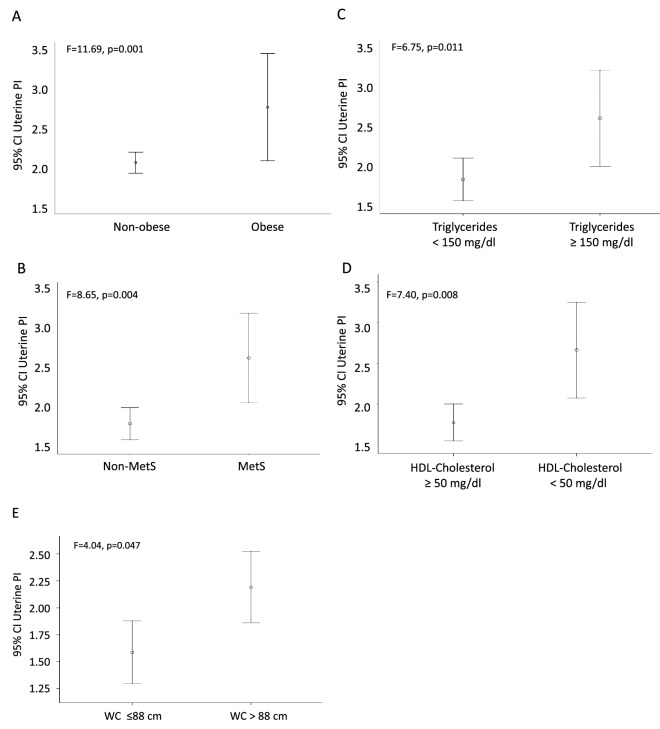
Differences in uterine artery pulsatility index (PI) in women with FSD according to obesity (BMI ≥ 30 kg/m^2^, panel **A**), metabolic syndrome (panel **B**), high triglyceride levels (≥ 150 mg/dL or taking lipid lowering drugs, panel **C**), low HDL-C levels (< 50 mg/dL, or taking lipid lowering drugs, panel **D**), and abdominal obesity (waist circumference > 88 cm, panel **E**). Data were adjusted for age age, years of and smoking habit. FSD = Female Sexual Dysfunction. BMI = body mass index. MetS = metabolic syndrome. TG = triglycerides. HDL-C = high density lipoprotein cholesterol. WC = waist circumference.

**Figure 3:**
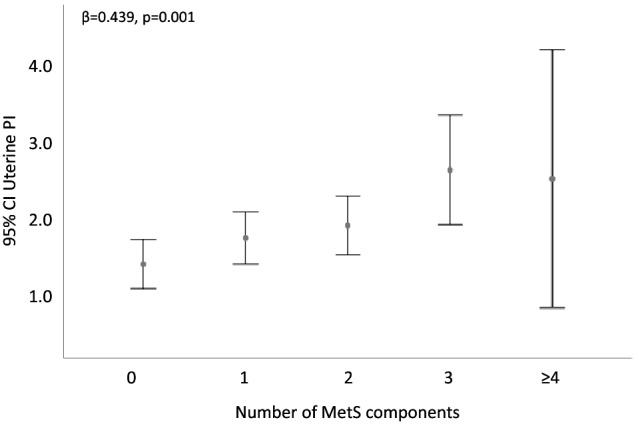
Association between uterine artery pulsatility index (PI) and the number of individual components of the metabolic syndrome (MetS), based on NCEP- ATP III criteria. Data were adjusted for age, years since menopause and smoking habit.

Similarly to clitoral PI, a significant positive association was found between uterine PI and several metabolic parameters (BMI, WC, insulin, HbA1c, and HOMA-IR index), but only in the subset of post-menopausal women (n = 65), in either the unadjusted or fully adjusted model (Table 3). In the same post-menopausal population, a significantly higher uterine artery PI value was demonstrated in obese women (BMI ≥ 30 kg/m^2^) as compared to non-obese ones (Fig. 2A). A similar higher uterine artery PI value was also found in post-menopausal women with MetS as compared to those without (Fig. 2B). In particular, among MetS factors, having triglyceride levels ≥ 150 mg/dL (or taking lipid lowering drugs) (Fig. 2C), or having HDL levels < 50 mg/dL (or taking lipid lowering drugs) (Fig. 2D), as well as having a WC > 88 cm (Fig. 2E) was associated with a significantly higher PI value. Noteworthy, uterine artery PI showed a stepwise increase as a function of increasing number of MetS components, even after adjusting for age, smoking habit, and years since menopause (β = 0.439, *p* = 0.001; Fig. 3).

**Table 3:** Associations between uterine pulsatility index (PI) and metabolic and psychological parameters in menopausal patients (N = 65).

	Uterine PI (in menopausal women)			
	Univariate analysis		Multivariate analysis*	
	Pearson’s r	*P*	β	*P*
**Clinical and metabolic parameters**				
BMI (kg/m^2^)	0.526	** < 0.0001**	0.511	** < 0.0001**
Waist circumference (cm)	0.491	** < 0.0001**	0.474	**0.001**
SBP (mm Hg)	0.091	0.485	0.083	0.578
DBP (mm Hg)	− 0.107	0.413	− 0.016	0.918
Fasting glycemia (g/L)	0.197	0.165	0.189	0.211
Total Cholesterol (mg/dL)	0.120	0.390	0.055	0.721
HDL Cholesterol (mg/dL)	− 0.250	0.074	− 0.260	0.088
Triglycerides (mg/dL)	0.181	0.203	0.202	0.192
LDL Cholesterol (mg/dL)	0.181	0.213	0.098	0.538
Fasting insulin (mUI/L)	0.416	0.018	0.481	**0.006**
HOMA-IR index	0.425	0.019	0.494	0.009
HbA1c (mmol/mol)	0.448	**0.003**	0.564	** < 0.0001**
**Psychological parameters**				
MHQ Total score	− 0.031	0.841	0.089	0.618
BUT-A global severity index (GSI)	0.436	**0.003**	0.652	** < 0.0001**
BUT-A weight phobia (WP)	0.314	0.038	0.543	**0.001**
BUT-A body image concern (BIC)	0.531	** < 0.0001**	0.669	** < 0.0001**
BUT-A avoidance (AV)	0.361	0.016	0.569	**0.001**
BUT-A compulsive self-monitoring (CSM)	0.070	0.653	0.185	0.270
BUT-A depersonalization (DEP)	0.430	**0.004**	0.684	** < 0.0001**
BUT-B positive symptom distress index (PSDI)	0.414	**0.006**	0.518	**0.001**
BUT-B positive symptom Total (PST)	0.095	0.548	0.271	0.131

*Data adjusted for age, smoking habit, and years since menopause. After 10% False Discovery Rate calculation, the cut-off for significant *p*-value was adjusted at < 0.038. Bold indicates statistical significance (*p* value < 0.038).

*BMI* body mass index, *BUT* Body Uneasiness Test, *HbA1c* glycated hemoglobin, *HDL* high-density lipoprotein, *HOMA-IR* homeostasis model assessment—insulin resistance, *LDL* low-density lipoprotein, *MHQ* Middlesex Hospital Questionnaire, *PI* pulsatility index.

In post-menopausal women, among the sexual parameters evaluated by FSFI, only the Desire domain was negatively associated with PI, but this association was not confirmed at multivariate analysis (not shown). Increased uterine PI was correlated with several items of the Body Uneasiness Test, such as BUT-A global measure (GSI) and BUT-B global distress measure (PSDI) in both univariate and multivariate analysis (Table 3). Uterine PI was also positively associated with several subdomains of BUT-A which explored weight phobia (WP), body image concerns (BIC), avoidance (AV), and depersonalization (DEP), even after adjusting for age, smoking habit, and years since menopause (Table 3); all these associations retained statistical significance also when introducing WC as a further covariate (β = 0.546, *p* = 0.002; β = 0.427, *p* = 0.013; β = 0.636, *p* = 0.002; β = 0.451, *p* = 0.009; β = 0.508, *p* = 0.005 for BUT-A GSI, WP, BIC, AV and DEP respectively).

## Discussion

In the present study we confirm that, in women consulting for sexual symptoms, clitoral artery pulsatility index (PI), a color Doppler ultrasound (CDU) hallmark of vascular resistance, was closely associated with BMI and several MetS factors. This study corroborates the potential role of clitoral PI in the assessment of cardiometabolic risk profiles in the sexological setting. Notably, concerning uterine PI, the above-mentioned associations were also observed, but only in the subset of post-menopausal women. An increased PI of either clitoral or uterine arteries was also associated with dissatisfaction with body image, as assessed by the Body Uneasiness Test.

A strong relationship between clitoral PI and BMI or MetS factors was originally demonstrated in a small pilot study (n = 71)^12^; hereby, we confirm further our previous findings in a larger study involving 230 women consulting for sexual symptoms. In particular, we found that clitoral PI increased as a function of WC, as well as of triglyceride, fasting insulin, and HOMA-IR levels, which are useful estimates for insulin resistance^32,33^. In the second part of the study, a subgroup of patients was evaluated with transvaginal CDU to investigate the uterine artery’s vascular parameters. Similarly to clitoral PI, uterine PI was independently correlated with several cardiometabolic risk factors, including increased BMI and WC, but only in the subset of post-menopausal women. Moreover, post-menopausal women affected by obesity and MetS displayed significantly higher uterine PI values, when compared to non-obese or non-MetS women, respectively. Uterine PI also showed a stepwise increase as a function of the increasing number of MetS parameters. These data suggest that uterine vascularization, which is easily explored by transvaginal CDU, is prone to specific changes caused by cardiometabolic disorders, as already observed in the clitoral district^12^. Therefore, uterine PI, as already proposed for clitoral PI, might be a mirror of CV health in women seeking medical care for sexual concerns, at least in the post-menopausal period. This is in line with previous studies conducted in women affected by PCOS, reporting that uterine PI was not only higher in overweight than in normal weight PCOS subjects, but it was also strongly correlated with the LDL-C/HDL-C ratio, a reliable predictor of CV risk^34,35^. Conversely, regarding lipid profile, we only found a close association between clitoral PI and triglyceride level, whilst uterine PI only showed a tendency towards a negative association with HDL cholesterol. Differences between the two study populations (PCOS vs. post-menopausal women) in terms of CV risk could account for the milder association that we observed in our study. However, similarly to what has been observed for clitoral PI, uterine PI appeared to be positively correlated to several parameters linked to glucose homeostasis and insulin resistance, specifically fasting insulin, HbA1c and HOMA-IR. Similar results were reported in a retrospective study of 155 women with pre-gestational diabetes, in which a significant association emerged between uterine PI and HbA1c^36^.

It is worth noting that uterine CDU is already a widespread and workable diagnostic technique in the obstetric and gynecological field. Evaluation of uterine blood flow has been used for decades in pregnancy as an index of placental vascularization, in order to predict the risk of fetal growth restriction and pre-eclampsia^14–16^, and more recently in the field of assisted reproductive technology (ART) to evaluate fertility outcomes^37–40^. In this context, an increased vascular resistance of the uterine artery has been correlated to a reduced endometrial flow and reduced success rate in ART outcomes^39^. Our work suggests a novel application of uterine CDU as an indirect, non-invasive evaluation of cardiometabolic profile, especially in the post-menopausal phase during the sexological work-up.

In other districts, such as the cerebral^41^ and the renal^42^, as well as in the aforementioned uterus-placental one^37^, an increased PI has been directly related to micro-arteriosclerosis processes. In addition, our study partially contributes to filling a gender gap in sexual medicine. It is well-established that CDU of penile arteries has a relevant role in identifying atherogenic ED and adverse CV and metabolic profiles in men^11,43^. However, while preclinical evidence suggests that the same mechanisms underpin male and female genital arousal disorders in metabolic diseases. the impact of MetS-related endothelial dysfunction on male sexuality, in particular in the development of ED, is well established^44–46^, while its role in women is likely milder, but also still under-investigated^47^. In a male rabbit model, high fat diet-induced Mets was related to modifications of molecular markers underlying nitric-oxide-cGMP-mediated relaxant pathways in penile tissue, thus inducing ED^44–46^. Accordingly, relevant histomorphological alterations of the clitoris have been observed in diabetic women, as compared to non-diabetic ones^48^ with degeneration of smooth muscle cells being significantly correlated with CV risk^49^.

In the present study, we found an inverse association between not only clitoral^12^, but also uterine PI, and several psychologic parameters, especially those related to dissatisfaction towards one’s general body image and genital area. Clitoral and uterine PI correlated with body uneasiness explored by the global BUT-B and the PSDI (Positive Symptom Distress Index) score. A correlation between uterine PI and BUT-A Global Severity Index and subdomains Weight Phobia, Body Image Concerns, Avoidance, and Depersonalization was also observed. These data suggest that, in women with sexual symptoms, a worse metabolic profile may negatively influence one’s body image, acting through a poor physical condition, as already observed in women with metabolic disorders^50–52^. In addition, a worse genito-pelvic haemodynamic function could result in an excessive focus on one’s body, especially on those parts directly involved in the sexual response^18^, such as the genitals.

Furthermore, clitoral PI increased as a function of the MHQ score related to somatized anxiety symptoms (MHQ-S). This observation is consistent with our previous work^12^, further suggesting that an increased vascular resistance (due to a dysfunctional vascular bed in subjects with an unfavorable metabolic profile) could lead to performance anxiety and body hypervigilance^53,54^. Vice versa, the opposite may occur, with anxiety-mediated hyperactivation of the sympathetic nervous system^55,56^.

In contrast with our previous study^12^, no significant associations were observed between the clitoral PI and sexual function as assessed by the FSFI total and subdomain scores. Since the present data are representative of a much larger sample, they support the idea that the FSFI may not be an accurate tool to detect and ascertain the organic component(s) of FSD^57^. Indeed, the use of psycho-sexual questionnaires instead of color Doppler ultrasound directly targeting the genital vascular district was hypothesized to be one of the main reasons accounting for the lack of conclusive clinical evidence on the association between CV and sexual health in women^47^. In this perspective, in our population of women seeking medical care for sexual concerns, hemodynamic parameters, such as PI, might lead to the screening for vascular alterations, as compared with indirect, subjective measures such as the FSFI.

Our study presents several limitations. First of all, the sample is heterogeneous, composed of patients with different clinical, pharmacological and gynecological histories. Second, the study was conducted in women consulting for sexual symptoms, limiting the generalization of the results. In addition, CDU is a dynamic and operator-dependent method, and uterine CDU evaluation can be influenced by gynecological conditions such as fibrosis or adenomyosis. However, we set our CDU protocol by avoiding the vessels close to the uterine abnormalities. Furthermore, parameters related to the partner and the dyadic relationship were not included^19,58^. Clinicians should assess sexual function concerning both partners and encompassing several dimensions like sexual satisfaction and perceived sexual interest in a patient’s partner. This may involve an interdisciplinary approach. A meta-analysis and systematic review found an association between sexual dysfunction in men partnered with women with sexual problems, especially in the domains of erectile and ejaculatory function^59^.

Finally, we have to recognize the lack of a longitudinal analysis investigating changes in cardiometabolic risk factors and the lack of a healthy control group as important limitations.

## Conclusions

In conclusion, increased vascular resistance, not only in clitoral but also in uterine arteries, is associated with cardiometabolic risk factors and body image concerns in women suffering from sexual symptoms. CDU of these genital districts may be thus proposed as an accessible method for the evaluation of cardiometabolic health in women counselling for sexual dysfunctions. If further confirmed in different populations of women, other than FSD ones, our data could suggest CDU, a common examination method widely used in gynecological practice, as a useful tool for an identification—and possible correction—of cardiometabolic risk factors. However, longitudinal studies are required to clarify whether increased clitoral and/or uterine artery PI may predict CV events.

## Supplementary Information

Supplementary Information 1.

Supplementary Information 2.

Supplementary Information 3.

## Data Availability

The datasets generated during and/or analysed during the current study are available from the corresponding author upon reasonable request.
