# Supplementary material for: Cardiometabolic risk is unraveled by color Doppler ultrasound of the clitoral and uterine arteries in women consulting for sexual symptoms
Source: Sci Rep. 2021 Sep 22;11:18899. doi: 10.1038/s41598-021-98336-7 (PMC8458448; doi:10.1038/s41598-021-98336-7)
Supplement: Supplementary file 1 — Supplementary Information 1. [file 41598_2021_98336_MOESM1_ESM.docx]

|  | **Total sample**  **N=230** | **Post-menopausal**  **N=114** | **Pre-menopausal**  **N=116** | **P** |
| --- | --- | --- | --- | --- |
| ***Clinical history*** | | | | |
| Age (years) | 43.1±12.9 | 55.9±7.1 | 35.4±9.0 | **<0.0001** |
| Menopause, % (n) | 49.9% (114) | - | - | - |
| Menopause, Surgical, % (n) | 5.0% (6) | - | - | - |
| Stable relationship, % (n) | 89.9% (207) | 87.5% (96) | 89.3% (107) | 0.674 |
| Current smoking habit, % (n) | 19.0% (44) | 20.0% (22) | 15.0% (18) | 0.378 |
| Physical activity, % (n) | 33.8% (78) | 43.8% (48) | 43.0% (52) | 1.000 |
| Parity, % (n) | 14.3% (33) | 72.0% (79) | 45.0% (54) | **0.026** |
| Waist circumference (cm) | 93.1±16.6 | 96.1±14.57 | 91.55±17.91 | 0.059 |
| BMI (kg/m^2^) | 24.9±6.1 | 25.8±5.55 | 24.42±6.60 | 0.084 |
| Cardiovascular diseases, % (n) | 3.0% (7) | 2.9% (3) | 3.0% (4) | 1.000 |
| Diabetes mellitus, % (n) | 4.3% (10) | 5.8% (6) | 4.1% (5) | 1.000 |
| Dyslipidemia, % (n) | 15.8% (36) | 19% (17) | 7% (8) | **0.004** |
| Hypertension, % (n) | 18.5% (43) | 24.3% (27) | 6% (7) | **<0.0001** |
| Specific medications |  |  |  |  |
| Hypoglycemic drugs, % (n) | 6.7% (15) | 8.3% (9) | 5.4%(6) | 1.000 |
| Lipid-lowering drugs, % (n) | 5.8% (13) | 10.2% (11) | 1.5%(2) | **0.006** |
| Antihypertensive drugs, % (n) | 15.4% (35) | 27.8% (30) | 5.4%(5) | **<0.0001** |
| Psychiatric drugs, % (n) | 23.3% (53) | 25% (27) | 24.2%(26) | 0.757 |
| Urinary or gynecologic infections  (actual or in the past 3 months), % (n) | 56.5% (130) | 25% (27) | 32.8%(103) | 0.308 |
| Urinary or gynecologic diseases and infections (in the past), % (n) | 52.2% (120) | 51.9% (59) | 50.8% (61) | 0.785 |
| Endometriosis, % (n) | 6.1% (14) | 2.9% (3) | 6.6% (9) | 0.234 |
| PCOS, % (n) | 5.5% (13) | 1.0% (1) | 7.4% (12) | **0.023** |
| Oral Contraception, % (n) | 16.9% (39) | 0% | 32.5% (39) | **<0.0001** |
| Hormonal Replacement Therapy, % (n) | 9.2% (21) | 19.1% (21) | 0% | **0.003** |
| Pelvic Surgery, % (n) | 26.9% (62) | 41.3% (45) | 21% (17) | **0.001** |
| Breast Surgery, % (n) | 8.6% (20) | 15.7% (17) | 5% (3) | **0.012** |
| Other Surgery , % (n) | 30.0% (69) | 49.1% (54) | 35.4% (15) | 0.059 |
| Oncologic diseases, % (n) | 11.7% (30) | 24.8% (27) | 2.5% (3) | **<0.0001** |
| Breast Cancer, % (n) | 1.6% (4) | 3.7% (3) | 1.0% (1) | **<0.0001** |
| Psychiatric diseases, % (n) | 33.3% (77) | 34.3% (37) | 33.3% (40) | 0.390 |
| Neurological diseases, % (n) | 1.8% (4) | 5.6% (3) | 0.9% (1) | 0.065 |
| ***Metabolic parameters*** | | | | |
| Systolic blood pressure (mm Hg) | 120.00  [110.00-130.00] | 125.00  [95.00-170.00] | 117.50  [89.00-160.00] | **0.001** |
| Diastolic blood pressure (mm Hg) | 75.00  [70.00-80.00] | 79.50  [55.00-105.00] | 50.00  [70.00-100.00] | 0.067 |
| Fasting glucose (g/L) | 0.90±0.14 | 0.95±0.24 | 0.90±0.23 | 0.096 |
| Fasting insulin (mU/L) | 9.20±8.91 | 9.75±7.67 | 10.47±10.60 | 0.671 |
| HbA1c (mmol/mol) | 36.01±5.48 | 37.97±7.47 | 35.06±6.25 | **0.013** |
| Total Cholesterol (mg/dl) | 201.90±38.08 | 218.19±36.72 | 192.81±34.75 | **<0.0001** |
| HDL Cholesterol (mg/dl) | 63.65±15.59 | 66.42±18.21 | 62.79±16.17 | 0.135 |
| LDL Cholesterol (mg/dl) | 119.38±32.37 | 130.61±32.15 | 112.96±29.12 | **<0.0001** |
| Triglycerides (mg/dl) | 80.00  [60.25-112.00] | 90.00  [66.00-126.50] | 70.50  [55.00-107.50] | **0.002** |

**Supplementary Table 1a.** Baseline characteristics of the sample (N=230), considered as a whole or after stratification according to the menopausal status: clinical history and metabolic parameters. Data are expressed as mean ± SD when normally distributed, median (quartile) when not normally distributed, and percentage when categorical. P values are derived from multivariate analysis, after adjusting for age. Bold indicates statistically significant difference (P < 0.038) between the 2 groups.

BMI= body mass index. Hba1c= glycated hemoglobin. HDL= high-density lipoprotein. LDL= low-density lipoprotein. PCOS= polycystic ovary syndrome.
