# Supplementary material for: Cardiometabolic risk is unraveled by color Doppler ultrasound of the clitoral and uterine arteries in women consulting for sexual symptoms
Source: Sci Rep. 2021 Sep 22;11:18899. doi: 10.1038/s41598-021-98336-7 (PMC8458448; doi:10.1038/s41598-021-98336-7)
Supplement: Supplementary file 2 — Supplementary Information 2. [file 41598_2021_98336_MOESM2_ESM.docx]

|  | **Total sample**  **N=230** | **Post-menopausal**  **N=114** | **Pre-menopausal**  **N=116** | **P** |
| --- | --- | --- | --- | --- |
| ***Psycho-sexual parameters*** | | | | |
| FSFI Total score | 20.6  [11.9 – 26.3] | 18.5  [11.2 – 24.7] | 22.4  [12.5 – 28.3] | **0.034** |
| FSFI Desire | 2.4 [1.8 – 3.6] | 2.4 [1.2– 3.0] | 2.4 [1.8 – 4.2] | **0.001** |
| FSFI Arousal | 2.7 [1.5 – 4.5] | 2.7 [1.5–4.2] | 3.3 [1.8 – 4.8] | 0.113 |
| FSFI Lubrication | 3.6 [1.5 – 5.4] | 3.3 [1.3 – 4.7] | 4.2 [1.9– 5.6] | 0.017 |
| FSFI Orgasm | 3.6 [1.2 – 5.2] | 3.2 [1.6 – 4.7] | 3.2 [1.2 – 5.2] | 0.859 |
| FSFI Satisfaction | 3.6 [2.0 – 5.2] | 3.6 [1.2 – 5.1] | 3.8 [2.0 – 5.2] | 0.161 |
| FSFI Pain | 3.6 [1.2 – 6.0] | 3.6 [1.8 – 0.6] | 3.6 [1.6 – 6.0] | 0.071 |
| FSFI pathological total score % (n) | 92.2% (212) | 91.2% (104) | 93.1% (108) | **0.014** |
| MHQ Total score | 35.5  [26.0 – 46.0] | 38.0  [29.0 – 49.0] | 37.0  [27.0 – 46.0] | 0.223 |
| FSDS-R score | 19.0  [7.0 - 33.0] | 25.5  [12.0 – 36.2] | 21.0  [7.0 – 33.0] | 0.146 |
| FSDS-R pathological score % (n) | 64.8% (149) | 61.4% (70) | 68.1% (79) | 0.232 |
| FSFI and FSDS-R pathological scores % (n) | 58.3% (134) | 56.1% (64) | 60.3% (70) | 0.866 |
| BUT-A global severity index (GSI) | 0.7 [0.4 – 1.5] | 0.8 [0.4 – 1.6] | 0.8 [0.3 – 1.8] | 0.882 |
| BUT-A weight phobia (WP) | 1.1 [0.5 – 2.4] | 1.1 [0.8 – 2.3] | 1.1 [0.6 – 2.4] | 0.779 |
| BUT-A body image concern (BIC) | 1.0 [0.5 – 1.9] | 1.0 [0.5 – 2.0] | 1.0 [0.4 – 2.2] | 0.583 |
| BUT-A avoidance (AV) | 0.2 [0.0 – 0.8] | 0.3 [0.0 – 1.1] | 0.2 [0.0 – 0.8] | 0.183 |
| BUT-A compulsive self-monitoring (CSM) | 0.6 [0.2 – 1.2] | 0.6 [0.4 – 1.1] | 0.8 [0.2 – 1.4] | 0.436 |
| BUT-A depersonalization (DEP) | 0.3 [0.0 – 0.8] | 0.3 [0.0 – 0.8] | 0.3 [0.0 – 0.9] | 0.573 |
| BUT-B positive symptom Total (PST) | 9.0 [5.0 – 17.0] | 8.0 [5.0 – 17.0] | 9.5 [7.0 – 16.0] | 0.469 |
| BUT-B positive symptom distress index (PSDI) | 2.0 [1.6 – 1.8] | 2.0 [1.4 – 3.1] | 2.0 [1.6 – 1.8] | 0.906 |
| ***CDU parameters*** | | | | |
| Clitoral PI | 1.6±0.7 | 1.7±0.8 | 1.5±0.6 | 0.767 |

**Supplementary Table 1b.** Baseline characteristics of the sample according to post- vs. pre-menopausal status: psycho-sexual parameters. Data are expressed as median (quartile). P values are derived from multivariate analysis, after adjusting for age. Bold indicates statistically significant difference (P < 0.038) between the 2 groups.

BUT= Body Uneasiness Test. FSDS-R= Female Sexual Distress Scale-Revised. FSFI= Female Sexual Function Index. MHQ= Middlesex Hospital Questionnaire. CDU= color Doppler ultrasound. PI= pulsatility index.
