# Supplementary material for: Cardiometabolic risk is unraveled by color Doppler ultrasound of the clitoral and uterine arteries in women consulting for sexual symptoms
Source: Sci Rep. 2021 Sep 22;11:18899. doi: 10.1038/s41598-021-98336-7 (PMC8458448; doi:10.1038/s41598-021-98336-7)
Supplement: Supplementary file 3 — Supplementary Information 3. [file 41598_2021_98336_MOESM3_ESM.docx]

|  | **Total sample (subgroup)**  **N= 164** | **Post-menopausal**  **N=65** | **Pre-menopausal**  **N=99** | **P** |
| --- | --- | --- | --- | --- |
| ***Clinical history*** | | | | |
| Age (years) | 46.1±12.9 | 55.5±7.4 | 35.1±8.7 | **<0.0001** |
| Menopause, % (n) | 39.1% (65) | - | - | - |
| Menopause, Surgical, % (n) | 2.9% (5) | - | - | - |
| Stable relationship, % (n) | 89.9% (147) | 90.5% (59) | 86.6% (86) | 1.000 |
| Current smoking habit, % (n) | 19% (31) | 27.3% (18) | 13.7% (14) | 0.043 |
| Physical activity, % (n) | 32.4% (53) | 27.1% (17) | 36.2% (36) | 0.860 |
| Parity, % (n) | 51% (83) | 73.8% (48) | 36% (36) | **<0.0001** |
| Waist circumference (cm) | 93.15±16.65 | 96.4±14.74 | 90.8±17.62 | 0.053 |
| BMI (kg/m^2^) | 24.97±6.15 | 25.8±5.65 | 24.4±6.42 | 0.157 |
| Cardiovascular diseases, % (n) | 3.0% (5) | 3.1% (2) | 3.0% (3) | 1.000 |
| Diabetes mellitus, % (n) | 4.3% (7) | 5.1%(4) | 3.1% (3) | 0.705 |
| Dyslipidemia, % (n) | 10.4% (17) | 17.2% (11) | 6.0% (6) | **0.034** |
| Hypertension, % (n) | 12.8% (21) | 25.0% (16) | 5.0% (5) | **<0.0001** |
| Specific medications |  |  |  |  |
| Hypoglycemic drugs % (n) | 8.3% (14) | 11.4% (8) | 6.3% (6) | 0.250 |
| Lipid-lowering drugs % (n) | 11.1% (18) | 22.2% (14) | 3.2% (4) | **0.004** |
| Antihypertensive drugs % (n) | 17.8% (29) | 28.3% (19) | 9.3% (10) | **<0.0001** |
| Psychiatric drugs % (n) | 25.0% (41) | 27.4% (18) | 24.5% (24) | 0.173 |
| Urinary or gynecologic infections  (actual or in the past 3 months), % (n) | 32.7% (53) | 27.7% (17) | 36% (36) | 0.310 |
| Urinary or gynecologic diseases and infections (in the past), % (n) | 56.5% (93) | 59.0% (38) | 55.0% (54) | 0.628 |
| Endometriosis, % (n) | 6.1% (10) | 3.2% (2) | 8.0% (8) | 0.319 |
| PCOS, % (n) | 5.5% (9) | 1.6% (1) | 7.9% (8) | 0.156 |
| Oral Contraception, % (n) | 16.2% (27) | - | - | - |
| Hormonal Replacement Therapy, % (n) | 5.8% (9) | - | - | - |
| Pelvic Surgery, % (n) | 25.3% (41) | 31.3% (20) | 21.4% (21) | 0.196 |
| Breast Surgery, % (n) | 11.9% (19) | 24.2% (15) | 4.1% (4) | **<0.0001** |
| Other Surgery , % (n) | 44.4% (73) | 50.8% (50) | 39.4% (23) | 0.200 |
| Psychiatric diseases, % (n) | 27.4% (45) | 34.0% (22) | 23.0% (23) | 0.177 |
| Neurological diseases, % (n) | 1.5% (3) | 1.6% (1) | 2.0% (2) | 1.000 |
| ***Metabolic parameters*** | | | | |
| Systolic blood pressure (mm Hg) | 120.00  [110.00-130.00] | 125.00  [110.00-135.00] | 110.00  [110.00-125.00] | **0.002** |
| Diastolic blood pressure (mm Hg) | 75.00  [70.00-80.00] | 80.00  [70.00-80.00] | 65.00  [70.00-80.00] | **0.034** |
| Fasting glucose (g/L) | 0.93±0.16 | 0.93±0.11 | 0.87±0.16 | **0.014** |
| Fasting insulin (mU/L) | 9.20±8.91 | 8.92±6.95 | 9.29±10.05 | 0.842 |
| HbA1c (mmol/mol) | 35.03±5.26 | 37.18±4.40 | 34.93±6.09 | **0.033** |
| Total Cholesterol (mg/dl) | 204.90±38.11 | 217.72±35.68 | 190.72±35.75 | **<0.0001** |
| HDL Cholesterol (mg/dl) | 64.63±15.61 | 65.66±17.35 | 62.42±14.20 | 0.215 |
| LDL Cholesterol (mg/dl) | 121.22±25.47 | 131.21±31.96 | 111.60±30.20 | **<0.0001** |
| Tryglicerides (mg/dl) | 81.00  [60.00-112.00] | 94.00  [68.00-121.00] | 70.50  [55.00-99.75] | **0.007** |
| ***Psycho-sexual parameters*** | | | | |
| FSFI Total score | 18.8  [10.8 - 25.1] | 18.5  [11.2 - 24.7] | 22.6  [13.4 - 28.5] | 0.053 |
| FSFI Desire | 2.4 [1.2 – 3.6] | 2.4 [1.20-3.00] | 2.4 [1.95-4.20] | **0.005** |
| FSFI Arousal | 2.7 [1.2 – 4.2] | 2.7[1.5 -4.45] | 3.3 [1.8– 4.8] | 0.081 |
| FSFI Lubrication | 3.3 [1.2 – 4.8] | 3.3[1.2 – 4.8] | 4.2 [2.7 – 5.7] | 0.035 |
| FSFI Orgasm | 3.2 [1.6 – 4.8] | 3.2[1.6 – 4.6] | 3.2 [1.2 – 5.2] | 0.905 |
| FSFI Satisfaction | 3.6 [1.6 – 5.2] | 3.2[1.2– 5.2] | 4.0 [2.4 – 5.2] | 0.109 |
| FSFI Pain | 3.2 [1.2 – 5.2] | 3.6[0.2 – 5.2] | 3.6 [1.6 – 5.0] | 0.117 |
| MHQ Total score | 37.0  [29.0 - 47.5] | 40.0  [29.0 - 51.0] | 39.0  [29.0 - 48.0] | 0.647 |
| FSDS-R Total score | 21.0 [8.0 - 34.5] | 26.0 [12.2 - 36.7] | 21.5 [7.2 - 34.0] | 0.167 |
| BUT-A global severity index (GSI) | 0.7 [0.3 - 1.5] | 0.8 [0.4 - 1.6] | 0.8 [0.4 - 1.8] | 0.813 |
| BUT-A weight phobia (WP) | 1.0 [0.4 – 2.1] | 1.1 [0.6 – 2.3] | 1.2 [0.6 – 2.4] | 0.479 |
| BUT-A body image concern (BIC) | 0.9 [0.5 – 1.7] | 1.3 [0.4 – 2.0] | 1.0 [0.4 – 2.2] | 0.922 |
| BUT-A avoidance (AV) | 0.3 [0.0 – 1.0] | 0.3 [0.0 – 1.2] | 0.2 [0.0 – 1.0] | 0.342 |
| BUT-A compulsive self-monitoring (CSM) | 0.6 [0.2 – 1.0] | 0.6 [0.4 – 1.2] | 0.8 [0.4 – 1.6] | 0.274 |
| BUT-A depersonalization (DEP) | 0.3 [0.0 – 0.7] | 0.3 [0.2 – 0.7] | 0.3 [0.0 – 1.0] | 0.684 |
| BUT-B positive symptom total (PST) | 8.0 [5.0 – 17.0] | 9.0 [4.0 – 17.0] | 10.5 [7.0 – 17.0] | 0.459 |
| BUT-B positive symptom distress index (PSDI) | 2.0 [1.5 – 3.2] | 2.0 [1.5 – 2.6] | 2.0 [1.7 – 2.9] | 0.699 |
| ***CDU parameters*** | | | | |
| Uterine PI | 2.0±0.9 | 1.9±0.9 | 2.1±0.9 | 0.218 |

**Supplementary Table 2.** Baseline characteristics of the subgroup of patients (N=164) on which transvaginal color Doppler ultrasound (CDU) with evaluation of uterine artery pulsatility index (PI) was performed, considered as a whole or after stratification according to the menopausal status: clinical history, metabolic parameters, and psycho-sexual parameters. Data are expressed as mean ± SD when normally distributed, median (quartile) when not normally distributed, and percentage when categorical. P values are derived from multivariate analysis, after adjusting for age. Bold indicates statistically significant difference (P < 0.038) between the 2 groups.

BMI= body mass index. BUT= Body Uneasiness Test. FSDS-R= Female Sexual Distress Scale-Revised. FSFI= Female Sexual Function Index. Hba1c= glycated hemoglobin. HDL= high-density lipoprotein. LDL= low-density lipoprotein. MHQ= Middlesex Hospital Questionnaire. PCOS= polycystic ovary syndrome. CDU= color Doppler ultrasound. PI= pulsatility index.
